# Key Concepts for assessing claims about treatment effects and making well-informed treatment choices

**DOI:** 10.12688/f1000research.16771.2

**Published:** 2019-01-23

**Authors:** Andrew David Oxman, Iain Chalmers, Astrid Austvoll-Dahlgren, Informed Health Choices group

**Affiliations:** 1 Centre for Informed Health Choices, Norwegian Institute of Public Health, Oslo, Norway; 2 University of Oslo, Oslo, Norway; 3 James Lind Initiative, Oxford, UK; 4 Regional Centre for Child and Adolescent Mental Health, Eastern and Southern Norway, Oslo, Norway

**Keywords:** concepts, critical thinking, critical appraisal, causal inference, treatment claims, informed decision making, epistemology

## Abstract

**Background**: The Informed Health Choices (IHC) Key Concepts are standards for judgement, or principles for evaluating the trustworthiness of treatment claims and treatment comparisons (evidence) used to support claims, and for making treatment choices. The list of concepts provides a framework, or starting point, for teachers, journalists and other intermediaries for identifying and developing resources (such as longer explanations, examples, games and interactive applications) to help people to understand and apply the concepts. The first version of the list was published in 2015 and has been updated yearly since then. We report here the changes that have been made from when the list was first published up to the current (2018) version.

**Methods**: We developed the IHC Key Concepts by searching the literature and checklists written for the public, journalists, and health professionals; and by considering concepts related to assessing the certainty of evidence about the effects of treatments. We have revised the Key Concepts yearly, based on feedback and suggestions; and learning from using the IHC Key Concepts, other relevant frameworks, and adaptation of the IHC Key Concepts to other types of interventions besides treatments.

**Results**: We have made many changes since the Key Concepts were first published in 2015. There are now 44 Key Concepts compared to the original 32; the concepts have been reorganised from six to three groups; we have added higher-level concepts in each of those groups; we have added short titles; and we have made changes to many of the concepts.

**Conclusions**: The IHC Key Concepts have proven useful in designing learning resources, evaluating them, and organising them. We will continue to revise the IHC Key Concepts in response to feedback. We welcome suggestions for how to do this.

## Background

You cannot make informed decisions without information. For decisions about actions to improve or maintain the health of individuals or communities (‘treatments’) to be well-informed and not misinformed, you need reliable information about the effects of treatments. Unfortunately, we are bombarded with claims about the benefits and harms of treatments, many of which are not reliable. Therefore people need to learn how to distinguish reliable from unreliable claims.

Unreliable claims about the benefits and harms of treatments are made in the mass media and social media, as well as in personal communications with family, friends, quacks, and health professionals ^1– 10^. They are made by governments, celebrities, journalists, advertisers, researchers, gurus, aunts, and uncles. They include claims about medicines, surgery and other types of “modern medicine”; lifestyle changes, such as changes in what you eat or how you exercise; herbal remedies and other types of “traditional” or “alternative medicine”; public health and environmental interventions; and changes in how healthcare is financed, delivered, and governed.

Many, if not most people are unable to assess the reliability of these claims. For example, in a survey of a random sample of Norwegian adults, we found that less than 20% of respondents recognized that lung cancer can be associated with drinking alcohol but not necessarily caused by it ^11^. This mirrors misleading claims that are commonly made in the media. For instance, stories about coffee frequently use language suggesting that cause and effect has been established, such as “coffee can kill you”, when reporting on associations that have been found between drinking coffee and various health outcomes ^12^. Personal experiences (anecdotes) are often used as a basis for treatment claims, and people are more likely to trust anecdotes than research. For example, surveys in the UK have shown that only about one third (37%) of the public trust evidence from medical research, while about two thirds (65%) trust the experiences of friends and family ^13^. In addition, anecdotes often exaggerate the alleged benefits of treatments (for cancer, for example) and ignore or downplay harms ^14^. At the same time, people in need or desperation hope that treatments will work and ignore potential harms.

Consequences of people’s inability to assess the reliability of treatment claims include overuse of ineffective and sometimes harmful treatments and underuse of effective treatments, both of which result in unnecessary suffering and waste ^15, 16^. For example, billions of dollars are wasted on alternative medicine and nutritional supplements for which there is no reliable evidence of benefits ^17, 18^. At the same time, millions of children die unnecessarily, in part because their parents do not seek and use effective treatments that are available to them ^19, 20^, and they don’t trust reliable claims about effective preventive treatments such as effective vaccines ^21^.

To address this problem, the Informed Health Choices (IHC) group is developing and evaluating resources to help people learn how to assess the trustworthiness of treatment claims and make well-informed decisions about treatments ^22, 23^. The first step in this work was to identify the key concepts that people need to understand and apply to do this ^24, 25^. We refer to these as the IHC Key Concepts. We review and update this list of concepts yearly. In this article we report the changes that we have made to the IHC Key Concepts since they were first published ^24^ and present the most recent (2018) version.

## Methods

The IHC Key Concepts are standards for judgment, or principles for evaluating the trustworthiness of treatment claims and treatment comparisons (research) used to support claims, and for making treatment choices. The list is intended to be relevant to people everywhere and to any type of treatment. Many of the concepts can be learned and used successfully by primary school children ^22, 26, 27^. Although we have developed and framed the Key Concepts to address treatment claims, people in other fields have also found them relevant. Work to adapt these concepts to apply to interventions in other fields is ongoing, including agricultural, economic, educational, environmental, international development, management, nutrition, policing, social welfare, and veterinary interventions.

The IHC Key Concepts are a starting point for developing learning resources to help people make judgements about the trustworthiness of claims about the effects of treatments (and other interventions), and to make well-informed decisions about treatments. They are also the basis for a database of multiple-choice questions that can be used to assess people’s abilities to assess treatment claims and make treatment choices ^28^. We have written the concepts and explanations in plain language. However, some of them may be unfamiliar and difficult to understand. The Key Concepts list is not intended to be a learning resource. It is a framework that can be used by teachers and others to identify and develop learning resources.

To develop the IHC Key Concepts, we first extracted all of the concepts addressed in Testing Treatments ^29^, a book that was written to promote more critical public assessment of claims about the effects of treatments. We then searched the literature for other relevant material, including books and checklists for the public, journalists, and health professionals ^24^. We also considered concepts related to making judgements about the certainty of evidence of the effects of treatments ^30^.

Our aim has been to include all concepts that are important for people to consider. At the same time, we have tried to minimise redundancy. We have organised the concepts in a way that we believe is logical, and we have sought feedback on this logic. The concepts are not organised based on how complex or difficult they are to understand and apply, or in the order in which they should be taught.

We have collected structured written feedback on the Key Concepts using a form with four questions ( Box 1). We initially obtained feedback from 29 members of an international advisory group ^24^. We have subsequently obtained responses to these questions at three workshops:

- Global Evidence Summit, Cape Town, South Africa, 14 September 2017
- Evidence Live, Oxford, UK, 20 June 2018
- 25 ^th^ Cochrane Colloquium, Edinburgh, UK, 17 September 2018

Box 1. **Questions used to elicit feedback on the Informed Health Choices (IHC) Key Concepts**

1. Are concepts included that should not be?

2. Are there important concepts that are missing?

3. Are the concepts organised in a logical way?

4. Do you have any other comments regarding the concepts?

In addition, we have sought feedback and suggestions from colleagues when we have presented the Key Concepts, and on our website. The Key Concepts are updated yearly, and once or twice each year the three authors review and discuss each new suggestion and feedback from workshops, and we reach a consensus on which, if any, changes to make to the Key Concepts. For each suggestion, we record our response and the rationale for it. We invite comments on planned revisions from the IHC group and others prior to finalising each update.

Three other sources of input have contributed to changes that we have made to the IHC Key Concepts. First, experience from developing learning resources and teaching has led to changes. For example, development of primary school resources ^31^ led to reorganising the concepts into three groups from the original six groups ^24^.

Second, we are reviewing related frameworks for critical thinking ^32^, including frameworks for teaching and learning critical thinking ^33– 37^; scientific reasoning, literacy, and thinking ^38– 41^; epistemic cognition ^42^; causal inference ^43^, problem solving ^44^, and meta-cognition ^45^; health literacy ^46– 48^; and evidence-informed decision making and evidence-based practice ^49– 51^. In addition to ideas for new concepts, this review has contributed to the development of lists of competences (required skills, knowledge, or capacity to do something) and dispositions (frequent and voluntary habits of thinking and doing) for thinking critically about treatments. We added these to the IHC Key Concept list in 2018.

Third, feedback from people who have adapted the IHC Key Concepts to claims and decisions about other types of interventions (such as educational, economic, and environmental interventions) has contributed to changes that we have made. This feedback contributed both to the decision to reorganise the Key Concept list in 2018 and to modifications of specific concepts.

## Results

The 2018 version of the IHC Key Concepts is the most recent version. It can be found as Supplementary File 1 and online ^52^. Before reporting the changes that we made in this version and the reasons for those changes, we summarise the changes that we made to the IHC Key Concepts in 2016 and 2017.

The first version of the IHC Key Concepts, published in 2015 ^24^, included 32 concepts in the following six groups:

- Recognising the need for fair comparisons of treatments
- Judging whether a comparison of treatments is a fair comparison
- Understanding the role of chance
- Considering all the relevant fair comparisons
- Understanding the results of fair comparisons of treatments
- Judging whether fair comparisons of treatments are relevant

In 2016 ^53^, we added two new concepts and reorganised the concepts into three groups. The two new concepts were:

- Unpublished results of fair comparisons may result in biased estimates of treatment effects.
- A lack of evidence is not the same as evidence of “no difference”.

The decision to reorganise the concepts into three groups grew out of our efforts to simplify the concepts and teach them to primary school children. The suggestion to use three groups - claims, comparisons, and choices - came from Matt Oxman, who had primary responsibility for writing the text for The Health Choices Book for primary school children ^54^. The book, which has been shown to be an effective learning resource in a randomised trial with over 10,000 children in Uganda, is a story in comic book format which introduces and explains 12 Key Concepts.

In 2017 ^55^, we added short titles for all the concepts and two new concepts:

- Peer-reviewed and published treatment comparisons may not be fair comparisons.
- Comparisons designed to evaluate whether a treatment can work under ideal circumstances may not reflect what you can expect under usual circumstances.

The suggestion to add the short titles came from Douglas Badenoch, the project manager for the Testing Treatments websites ^54^. The short titles were needed for the Critical thinking and Appraisal Resources Library (CARL) on the Testing Treatments - English website. CARL is a database of learning resources for teachers and others who are responsible for encouraging critical thinking about treatment claims ^56^. It contains over 500 open-access learning resources in a variety of formats, including text, audio, video, webpages, cartoons, and lesson materials. Each resource is relevant to at least one IHC Key Concept and CARL can be searched or browsed using the Key Concepts.

In the 2018 version ( Supplementary File 1), we merged two Key Concepts and added nine new concepts. We reorganised the concepts within each of the three main groups and added three subgroups to each of the three main groups of concepts. We also replaced all of the short titles and introduced emojis.

We removed the concept that “hope or fear can lead to unrealistic expectations about the effects of treatments” and incorporated this in the explanation of the concept “treatments may be harmful”. The explanation begins with “People often exaggerate the benefits of treatments and ignore or downplay potential harms.” We added: “Similarly, people in need or desperation hope that treatments will work and ignore potential harms.”

The nine new concepts were:

- We can rarely, if ever, be 100% certain about the effects of treatments.
- People often recover from illness without treatment.
- More data is not necessarily better data, whatever the source.
- It is rarely possible to know in advance who will benefit, who will not, and who will be harmed by using a treatment.
- Indirect comparisons of treatments can be misleading.
- Outcomes should be assessed reliably in treatment comparisons.
- Treatment comparisons may be sensitive to assumptions that are made.
- Verbal descriptions of treatment effects can be misleading.
- The problem and the treatment options being considered may not be the right ones.

We introduced three higher level concepts within each of the three groups of Key Concepts and reframed the titles of the three groups as shown in Box 2.

Box 2. **Higher-level concepts used to reorganise the Informed Health Choices (IHC) Key Concepts in 2018**

1. **Beware of treatment claims like these**

1.1 Beware of claims that are too good to be true.
1.2 Beware of claims based on faulty logic.
1.3 Beware of claims based on trust alone.

2. **Check the evidence from treatment comparisons**

2.1 Don’t be misled by unfair comparisons.
2.2 Don’t be misled by unreliable summaries of treatment comparisons.
2.3 Don’t be misled by how treatment effects are described.

3. **Make well-informed treatment choices**

3.1 What is the problem and what are the options?
3.2 Is the evidence relevant?
3.3 Do the advantages outweigh the disadvantages?

We did this in response to feedback that the organisation of concepts within the three main groups was not logical, and that having long lists of concepts was overwhelming. The subgroups of concepts, using these higher-level concepts, provides a more transparent logic for how the concepts are organised in each main group. Having just three higher level concepts for each group may also make it easier to get the gist of the concepts and make the list less overwhelming and easier to remember.

There were three reasons for changing the short titles used for each of the Key concepts. First, we had received feedback that the short titles were not consistent with some of the concepts and that some were not short; and it was difficult to come up with a short, catchy title that accurately reflected each concept. Second, we wanted short titles that were consistent with the new organisation of the concepts. Third, short titles that we were developing for posters and a website targeted at school children seemed to be a solution to this problem. We added emojis to make the poster and website that we are developing more appealing. When presenting these to colleagues and others, the emojis appeared to appeal across age groups and to reflect the content accurately, which also may help to convey the gist of the concepts. The full list of short titles for the Key Concepts and the emojis are shown in Box 3.

**Box 3.** Overview of the 2018 version of the Informed Health Choices (IHC) Key Concepts (short titles)

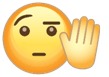 **1. Beware of treatment claims like these** We hear claims about the effects of treatments all the time. Many of these are not trustworthy. When you hear someone use one of these reasons to support a claim about the effects of a treatment, you should beware and ask where the evidence is.	
**1.1 Beware of claims that are too good to be true.** a) “100% safe!” b) “100% effective!” c) “100% certain!” **1.2 Beware of claims based on faulty logic.** a) “Treatment needed!” b) “It works like this!” c) “Associated with!” d) “Real world data!” e) “No comparison needed!” f) “A study shows!”	g) “Old is better!” h) “New is better!” i) “More is better!” j) “Early is better!” k) “Personalised medicine!” ** 1.3) Beware of claims based on trust alone.** a) “As advertised!” b) “It worked for me!” c) “Recommended by experts!” d) “Peer reviewed!”
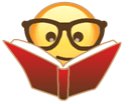 **2. Check the evidence from treatment comparisons** A treatment has to be compared to something else to know what would happen without the treatment. For treatment comparisons to be FAIR, the only important difference between comparison groups should be the treatments they receive. Unfair treatment comparisons and unsystematic summaries of treatment comparisons can be misleading. The way that treatment effects are described can also be misleading.	
**2.1 Don’t be misled by unfair comparisons!** a) Dissimilar comparison groups b) Indirect comparisons c) Dissimilar attention and care d) Dissimilar expectations or behaviours e) Dissimilar assessment of outcomes f) Unreliable assessment of outcomes g) Lots of people not followed-up h) Outcomes counted in the wrong group **2.2 Don’t be misled by unreliable summaries of treatment** **comparisons!** a) Unsystematic summaries	b) Selective reporting c) Unfounded assumptions **2.3 Don’t be misled by how treatment effects** **are described!** a) Just words b) Relative effects c) Average effects d) Few people or events e) Subgroup analyses f) Statistically significant g) No confidence interval h) No evidence
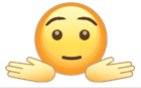 **3. Make well-informed treatment choices** Deciding what to do requires judgements about the relevance of the evidence, how important the good and bad outcomes are to you, and how sure you can be about the treatment effects.	
**3.1 What is the problem and what are the options?** a) What is your health problem and what are your options? **3.2 Is the evidence relevant?** a) What outcomes matter to you? b) Are the people (or animals) very different from you?	c) Are the treatments different from those available to you? d) Are the circumstances different from yours? **3.3 Do the advantages outweigh the disadvantages?** a) Do the advantages outweigh the disadvantages for you? b) How sure are you about the treatment effects?

### Other changes made to the IHC Key Concepts

In addition to adding 13 new Key Concepts and removing one since the first version was published in 2015, and reorganising the concepts, we have modified several of them. Most of these changes have been in response to suggestions to add new concepts when we concluded that it made more sense to incorporate the suggestion in an existing concept. These changes are summarised in Table 1.

**Table 1.** Changes made to IHC Key Concepts in response to suggestions.

Suggested addition	Key Concept that was modified	Change that was made
Analogies, such as drug class effects, and causal understanding of the body sometimes predict the direction but not the size of treatment effects.	Beliefs about how treatments work are not reliable predictors of the presence or size of actual effects of treatments	We added this to the explanation: And even if there is plausible evidence that a treatment works in ways likely to be beneficial, the size of any such treatment effect, and its safety, cannot be predicted. For example, most drugs in a class of heart medicines called beta-blockers have beneficial effects in reducing recurrence of heart attacks; but one of the drugs in the class – practolol – caused unpredicted serious complications in patients’ eyes and abdomens.
Replication	The results of single comparisons of treatments can be misleading	We clarified that this is addressed by adding “replications” to the explanation: Systematic reviews of these other comparisons (replications) may yield different results from those based on the initial studies, and these should help to provide more reliable and precise estimates of treatment differences.
Technology is always better.	New, brand-named, technologically impressive, or more expensive treatments may not be better than available alternatives	We added “technologically impressive” to the concept that new is not necessarily better.
Disease mongering	Earlier detection of ‘disease’ is not necessarily better	We put ‘disease’ in quotes. We also added “statistical risk of disease” to the explanation: People often assume that early detection of disease and ‘treating’ people who are at statistical risk of disease lead to better outcomes.
Regression to the mean	Personal experiences or anecdotes (stories) are an unreliable basis for assessing the effects of most treatments	We added the following to the explanation: One reason that personal experiences - including a series of personal experiences - are sometimes misleading is that experiences, such as pain, fluctuate and tend to return to a more normal or average level. This is sometimes referred to as "regression to the mean". For example, people often treat symptoms such as pain when they are very bad and would improve anyway without treatment. The same applies to a series of experiences. For example, if there is a spike in the number of traffic crashes someplace, traffic lights may be installed to reduce these. A subsequent reduction may give the appearance that the traffic lights caused this change. However, it is possible that the number of crashes would have returned to a more normal level without the traffic lights.
Common lay opinion is also not always right.	Opinions of experts or authorities do not alone provide a reliable basis for judging the benefits and harms of treatments	We added “like anyone else” to the explanation: Doctors, researchers, and patients – like anyone else - often disagree about the effects of treatments.
We can be misled by liking the expert or person who says something.	Opinions of experts or authorities do not alone provide a reliable basis for judging the benefits and harms of treatments	We addressed this suggestion in the explanation for this concept: Who makes a treatment claim, how likable they are, or how much experience and expertise they have are not a reliable basis for assessing how reliable their claim is.
Just because evidence is widely or easily accessible does not mean that it is trustworthy.	Peer-reviewed and published treatment comparisons may not be fair comparisons	We added this to the explanation: Similarly, just because a study is widely publicised does not mean that it is trustworthy.
Include nocebo effect	If possible, people should not know which of the treatments being compared they are receiving	We added this to the explanation: People in a treatment group may also experience harms (for example, more pain) because of their expectations (this is called a nocebo effect). And we added 'or worse' here: If individuals know that they are receiving a treatment that they believe is better or worse . . .
Contamination	People’s outcomes should be counted in the group to which they were allocated	We added the following to the explanation: “Contamination“ may lead to an underestimate of effect relative to what would have happened if everyone had received what was intended.
Evidence can change over time.	Reviews of treatment comparisons that do not use systematic methods can be misleading	We added up-to-date to the implication: Whenever possible, use up-to-date systematic reviews of fair comparisons inform decisions
Protocols	Reviews of treatment comparisons that do not use systematic methods can be misleading Unpublished results of fair comparisons may result in biased estimates of treatment effects	We added the following to the explanation for the first concept: To avoid these problems, systematic reviews of fair comparisons begin with protocols, which should be registered and searchable in registries such as Prospero. And we added the following to the explanation for the second concept: Selective reporting is an important reason why fair comparisons of treatments should have protocols that are registered and searchable in registries such as clinicaltrials.gov.
Short-term effects may not reflect long- term effects.	A systematic review of fair comparisons of treatments should report outcomes that are important	We added “short and long-term” to the first sentence of the explanation: A fair comparison may not include all outcomes - short and long- term - that are important to you. And we added this to the end of the explanation: Similarly, short-term effects may not reflect long-term effects.
Patient preference	Decisions about treatments should not be based on considering only their benefits	We added this to the explanation: The balance also depends on how much people value (how much weight they give to) the treatment advantages and disadvantages. Different people may value outcomes differently and sometimes make different decisions because of this.
The word ‘unlike’ is confusing. ‘Dissimilar’ would make more sense.	Don’t be misled by unfair comparisons	We had changed ‘dissimilar’ to ‘unlike’ because we thought that unlike is more likely to be understood by most English speakers, including children. It is also consistent with the idea of ‘comparing like with like’. However, based on the feedback we received, we changed unlike back to dissimilar.

### Suggestions that have been made when we concluded no change was needed

In addition to feedback from three workshops over the past two years, we have received 61 suggestions for revisions over the past three years. For many of these we concluded that no change was needed. Several suggestions were similar. We summarise these suggestions and our reasons for not making any changes in Table 2.

**Table 2.** Suggestions for which no changes were made to the IHC Key Concepts.

Suggestion	Related IHC Key Concepts	Reason for not making a change
Type 1 and type 2 errors	Small studies in which few outcome events occur are usually not informative and the results may be misleading The use of p-values may be misleading; confidence intervals are more informative Saying that a difference is statistically significant or that it is not statistically significant can be misleading	This suggestion is addressed by these concepts. In addition, this terminology may contribute to misleading interpretations of statistical significance.
Framing of effects	Relative effects of treatments alone can be misleading	While there is evidence that relative effects alone can be misleading ^57^, the effects of positive compared to negative framing are uncertain ^58^.
Data extrapolation	A systematic review of fair comparisons of treatments should report outcomes that are important A systematic review of fair comparisons of treatments in animals or highly selected groups of people may not be relevant The treatments evaluated in fair comparisons may not be relevant or applicable Comparisons designed to evaluate whether a treatment can work under ideal circumstances may not reflect what can be expected under usual circumstances.	This suggestion is addressed by these concepts.
Biased reporting	Don’t be misled by how treatment effects are described	This suggestion is addressed by these concepts.
It is not necessary to demonstrate what is true in order to demonstrate what is false.		This suggestion is not a useful concept for assessing the trustworthiness of treatment claims.
Natural course of disease	People often recover from illness without treatment	This suggestion is addressed in the explanation for this concept.
Heterogeneity or risk stratification	The results of single comparisons of treatments can be misleading Relative effects of treatments alone can be misleading Average differences between treatments can be misleading	This suggestion is addressed by these concepts.
Intuition	Opinions of experts or authorities do not alone provide a reliable basis for judging the benefits and harms of treatments	This suggestion is addressed by this concept.
Spill-over effects	A systematic review of fair comparisons of treatments should report outcomes that are important	This suggestion is addressed by this concept - to the extent that spill-over effects are an important consideration.
Where do I get reliable information?		This suggestion is outside the scope of the IHC Key Concepts.
Criteria of health information (parts of it are already included)		Other criteria that are used to assess health information - for example, readability - are outside the scope of the IHC Key Concepts.
Quality of systematic reviews	Reviews of treatment comparisons that do not use systematic methods can be misleading	This suggestion is addressed by this concept
It may be helpful to specify than advantages/ disadvantages may be different between patients, clinicians and policy makers.	Decisions about treatments should not be based on considering only their benefits	We have made clear in all of the concepts in the third group that the focus is on judgements made from ‘your’ perspective in the new short titles and the explanations. Although we have not specified that policymakers may have different perspectives than individual patients, this is implicit and can be included in learning-resources when this is relevant.
Systematic reviews currently described as a threshold of reliability but this isn't the case - many systematic reviews are not reliable and many other types of evidence can be reliable or better than nothing in certain contexts.	The results of single comparisons of treatments can be misleading Don’t be misled by unfair comparisons Reviews of treatment comparisons that do not use systematic methods can be misleading	Systematic reviews are not described as a threshold; they are described as the starting point for making judgements about the certainty of the evidence. These concepts explain why systematic reviews are needed and the need to assess the trustworthiness of treatment comparisons. They do not suggest that nothing is necessarily better than a single study, when that is the only evidence that is readily available.
Clear questions are necessary for fair comparisons.	The problem and the treatment options being considered may not be the right ones	This suggestion is relevant for researchers, not for people using research. We added the parallel concept that is relevant for people making decisions to the third group of concepts.
Treatments should be provided by someone with the necessary skills.	The treatments evaluated in fair comparisons may not be relevant or applicable	This suggestion is addressed by this concept.
Beware of manipulative use of language and pictures.	Verbal descriptions of treatment effects can be misleading	We incorporated this suggestion in the explanation for this new concept.
Having started and invested in a treatment doesn’t mean that it works and you should keep taking it.	Treatments may be harmful	This suggestion is similar to the concept that hope or fear can lead to unrealistic expectations about the effects of treatments, and does not warrant a separate concept. The concept about hope is now incorporated in the explanation for the concept that people often exaggerate the benefits of treatments and ignore or downplay potential harms.
Don’t be distracted by irrelevant information.	Verbal descriptions of treatment effects can be misleading	This suggestion is addressed by this new concept, which we have added.
Was the recommendation made by a group with an appropriate mix of skills and perspectives?		This suggestion is outside the scope of the IHC Key Concepts.
There should be something about the difference between slow and fast thinking.		This is not a concept. It is addressed as a competence - Recognise when to go from quick to slow thinking about treatment claims - and as a disposition - Go from fast to slow thinking before forming an opinion about a treatment claim, making a claim, or taking a decision
Not all treatments always feel comfortable.	A systematic review of fair comparisons of treatments should report outcomes that are important	This suggestion is addressed in the explanation for this concept
Uncertain about ‘personalised medicine’ as a claim, having never come across this	It is rarely possible to know in advance who will benefit, who will not, and who will be harmed by using a treatment	Claims about personalised medicine are widespread. And the concept that it is rarely possible to know who will benefit, who will not, and who will be harmed by a treatment is fundamental.
The ability to recognise or challenge claims that come from sources that are considered reliable		We added this as a competence: Communicate with others about the advantages and disadvantages of treatments
Be critical of the source of the claim.	Beware of claims based on trust alone	This is addressed by these concepts.
Some of these are true.	Beware of claims that seem too good to be true	We do not say that they are never true.
Unfair to compare interventions that are apples and oranges or chalk and cheese; e.g. by combining them in a meta-analysis	Unfair comparisons	This is implicitly a consideration for 'Unsystematic summaries' and could be added explicitly to the explanation. However, it is one of many considerations that could be added as concepts under 'unreliable summaries of comparisons'. It is outside of the scope of the IHC Key Concepts to go into that level of detail and we do not see a compelling argument for adding this specific consideration and not others that could be included in a checklist for assessing the reliability of a systematic review.

Suggested revisions to the IHC Key Concepts and responses 2016–2018 Click here for additional data file. Copyright: © 2019 Oxman AD et al. 2019 Data associated with the article are available under the terms of the Creative Commons Zero "No rights reserved" data waiver (CC0 1.0 Public domain dedication).

## Discussion

Up to now we have received much positive feedback, along with many suggestions for improvements, on the IHC Key Concepts, including positive feedback on the changes that we made in the 2018 version. Nonetheless, as can be seen from the results reported here, we have made many changes since the Key Concepts were first published in 2015. There are now 44 Key Concepts compared to the original 32; the concepts have been reorganised from six to three groups; we have added higher-level concepts within each of those groups; we have added short titles; and we have made changes to many of the concepts. We will continue to revise the IHC Key Concepts in response to feedback. Although we and others have found the concepts helpful since they were first published ^24^, we anticipate that there will still be ways in which they can be further improved. We welcome suggestions on ways of doing this.

The most common misunderstanding in the feedback we have received is that the Key Concepts list is a learning resource intended for people with no relevant research background. As noted in the Methods section, the list of Key Concepts serves as the basis for developing learning resources. It is not designed as a learning resource. It is a framework, or starting point, for identifying and developing learning resources.

Another common misunderstanding is that the Key Concepts are organised in the order in which they should be taught or learned. We have organised the Key Concepts logically by grouping them first in three groups and then within those three groups using higher-level concepts ( Box 2). This logic does not reflect the difficulty of the concepts or the order in which they should be learned.

When teaching the concepts, it may make sense to start with ones in the first group, followed by ones in the second group, followed by ones in the third group. However, it does not necessarily make sense to teach them in that order or in the order that they are organised within each group. For example, at least 24 of the Key Concepts can be understood and applied by primary school children ^31^, whereas other concepts are likely too difficult for primary school children to understand and use. Thus, it would obviously make sense to hop over those concepts when teaching primary school children.

Also, it is important not to try to teach or learn too much at one time. We initially tried teaching 24 Key Concepts to primary school children in one go, and found that was too much to teach in a single school term ^31^. Our efforts to teach IHC Key Concepts to both primary school children and their parents support our initial hypothesis that the time to start learning these concepts is in primary school - if not even younger ^59^. Ideally, these concepts should be taught and learned using a spiral curriculum ^60– 62^, that maps out what students should learn, where they should begin, and how they should progress to master these skills. Each cycle in a spiral curriculum reinforces what was learned previously while introducing new concepts. This can help teachers and students identify when milestones have been reached, build a foundation for later stages of learning, and guide the development of assessment tools and learning resources. We have not yet developed a spiral curriculum based on the IHC Key Concepts.

Decisions about the suggestions we have received have been based on logic and discussion. Four criteria have emerged from these discussions, which we will use explicitly in further developing the IHC Key Concepts. New Key Concepts have to:

- be within the scope of the IHC Key Concepts - standards for judgment, or principles for evaluating the trustworthiness of treatment claims and treatment comparisons (research) used to support claims, and to inform treatment choices
- address ways in which treatment claims and comparisons are frequently misleading or ways in which poorly informed decisions are taken
- be useful for people without a research background to use research, not just for researchers or for doing research
- overlap as little as possible with other Key Concepts

In addition to continuing to seek and review feedback and suggestions, we will further develop the Key Concepts by continuing to learn from using the IHC Key Concepts, other relevant frameworks, and adaptation of the IHC Key Concepts to other types of interventions. We also plan to summarise the evidence supporting each of the Key Concepts.

## Conclusions

The IHC Key Concepts have proven useful in designing learning resources, evaluating them, and organising them ^25^. The most recent version of the Key Concepts improves on previous versions by incorporating additional Key Concepts, organising the Key Concepts more logically and, we believe, making it easier to get the gist of the Key Concepts. Future improvements will be made based on feedback and suggestions, and ongoing evaluation.

## Data availability

The data referenced by this article are under copyright with the following copyright statement: Copyright: © 2019 Oxman AD et al.

Data associated with the article are available under the terms of the Creative Commons Zero "No rights reserved" data waiver (CC0 1.0 Public domain dedication). 

Dataset 1: Suggested revisions to the IHC Key Concepts and responses 2016-2018 https://dx.doi.org/10.5256/f1000research.16771.d223532 ^63^

## Supplementary material

Supplementary File 1: Informed Health Choices (IHC) Key Concepts 2018
