# Supplementary material for: Key Concepts for assessing claims about treatment effects and making well-informed treatment choices
Source: F1000Res. 2019 Jan 23;7:1784. Originally published 2018 Nov 12. [Version 2] doi: 10.12688/f1000research.16771.2 (PMC6290969; doi:10.12688/f1000research.16771.2)
Supplement: Supplementary file 2 [file f1000research-7-19687-s0001.tgz › 6752e950-fc5a-405a-b6d8-cc314aa0b4d8_2018_IHC.pdf]

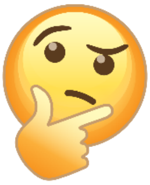

Key Concepts for assessing claims about treatment effects and making well informed treatment choices

Introduction

There are endless claims about treatments in the mass media, advertisements, and everyday personal communication. Some are true and some are false. Many are unsubstantiated: we do not know whether they are true or false. Unsubstantiated claims about the effects of treatments are often wrong. Consequently, people who believe and act on these claims suffer unnecessarily and waste resources by doing things that do not help and might be harmful, and by not doing things that do help.

In response to these challenges, we developed the IHC Key Concepts as the first step in the Informed Health Choices project, an initiative supported by the Research Council of Norway. The aim of the project is to help people make informed health choices.

A treatment is any intervention (action) intended to improve health, including preventive, therapeutic and rehabilitative interventions, and public health or health system interventions. Although we have developed and framed the Key Concepts to address treatment claims, people in other fields have also found them relevant; for example, for assessing claims about the effects of educational interventions or environmental measures. Work to adapt these concepts to other fields is ongoing.

The Informed Health Choices (IHC) Key Concepts

The concepts serve as the basis for developing learning resources to help people understand and apply the concepts when claims about the effects of treatments (and other interventions) are made, and when they make health choices.[1] They are also the basis for a database of multiple-choice questions that can be used for assessing people's ability to apply the IHC Key Concepts.[2]

The IHC Key Concepts serve as standards for judgment, or principles for evaluating the trustworthiness of treatment claims, comparisons, and choices. The list is intended to be universally relevant. The concepts can help people to:

1. Recognise when a **claim** about the effects of treatments has an untrustworthy basis
2. Recognise when evidence from **comparisons** of treatments is trustworthy and when it is not
3. Make well-informed **choices** about treatments

What's new?

We started to develop this list of concepts in 2013. We published the first version of the list in 2015 (original version), with 32 concepts in six groups. We published revised lists in October 2016 with 34 concepts in three groups and in 2017 with 36 concepts. The current list has 44 concepts in the same three groups. We have reorganised the concepts within each of the three main groups and added three subgroups to each of the three main groups of concepts. We did this partially in response to feedback that the organisation of concepts within the three main groups was not logical, and that having long lists of concepts was overwhelming. The subgroups of concepts provides a more transparent logic for how the concepts are organised in each main group. Having just three higher level concepts for each group may also make it easier to get the gist of the concepts, and make the list less overwhelming and easier to remember.

[1] Chalmers I, Oxman AD, Austvoll-Dahlgren A, et al. Key Concepts for Informed Health Choices: A framework for helping people learn how to assess treatment claims and make informed choices. BMJ Evid Based Med 2018; 23:29-33.

[2] Austvoll-Dahlgren A, Semakula D, Nsangi A, et al. Measuring ability to assess claims about treatment effects: the development of the 'Claim Evaluation Tools'. BMJ Open 2017; 7:e013184.

Competences and dispositions

In addition to modifying the Key Concepts, this year we have added lists of competences (required skills, knowledge, or capacity to do something) and dispositions (frequent and voluntary habits of thinking and doing) for thinking critically about treatments.

How we developed this list of Key Concepts

We developed the IHC Key Concepts by searching the literature and checklists written for the public, journalists, and health professionals, and by considering concepts related to assessing the certainty of evidence about the effects of treatments.[3] We have tried to include all concepts that are important for people to consider when they assess treatment claims and make health choices. At the same time, we have tried to limit the number of concepts by minimising redundancy. We have organised the concepts in a way that makes sense to us and others who have provided feedback. They are not organised based on how complex or difficult they are to understand and apply, or in the order in which they should be learned. That is something we plan to do in the future.

Although we have written the concepts and explanations in plain language, some of them may be unfamiliar and difficult to understand. The list is not designed as a teaching tool. It is a framework, or starting point, for teachers, journalists and other intermediaries for identifying and developing resources (such as longer explanations, examples, games and interactive applications) to help people to understand and apply the concepts.

When will the list of concepts next be updated?

The list is subject to yearly review by a working group at the Centre for Informed Health Choices in Oslo. Astrid Austvoll-Dahlgren, Iain Chalmers, and Andy Oxman led the development of the original list of IHC Key Concepts and have amended it in the light feedback and suggestions since then. They are responsible for the final decisions about amendments and additions. Many other people have contributed to this work, including other members of the IHC team and people from around the world with different types of expertise. The next update will be made available in October 2019. Please send any comments or suggestions to: contact@informedhealthchoices.org.

Where you can find more information

More information about the IHC Key Concepts, their development, and their use can be found on the IHC website.

Suggested citation: Oxman AD, Chalmers I, Austvoll-Dahlgren A, and the Informed Health Choices Group. Key Concepts for assessing claims about treatment effects and making well-informed treatment choices. Version: 2018. https://www.informedhealthchoices.org/wp-content/uploads/2018/10/Key-Concepts-2018-edition.pdf.

[3] Austvoll-Dahlgren A, Oxman AD, Chalmers I, et al. Key concepts that people need to understand to assess claims about treatment effects. J Evid Based Med 2015; 8:112-25.

Overview of the IHC Key Concepts (short titles)

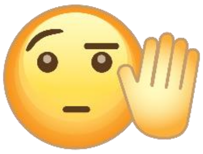

1. Beware of treatment claims like these

We hear claims about the effects of treatments all the time. Many of these are not trustworthy. When you hear someone use one of these reasons to support a claim about the effects of a treatment, you should beware and ask where the evidence is.

1.1 Beware of claims that are too good to be true.

a) "100% safe!"
b) "100% effective!"
c) "100% certain!"

1.2 Beware of claims based on faulty logic.

a) "Treatment needed!"
b) "It works like this!"
c) "Associated with!"
d) "Real world data!"
e) "No comparison needed!"
f) "A study shows!"
g) "Old is better!"
h) "New is better!"
i) "More is better!"
j) "Early is better!"
k) "Personalised medicine!"

1.3 Beware of claims based on trust alone.

a) "As advertised!"
b) "It worked for me!"
c) "Recommended by experts!"
d) "Peer reviewed!"

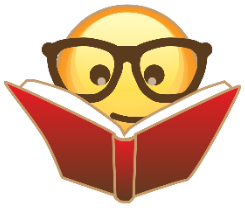

2. Check the evidence from treatment comparisons

A treatment has to be compared to something else to know what would happen without the treatment. For treatment comparisons to be FAIR, the only important difference between comparison groups should be the treatments they receive. Unfair treatment comparisons and unsystematic summaries of treatment comparisons can be misleading. The way that treatment effects are described can also be misleading.

2.1 Don't be misled by unfair comparisons!

a) Dissimilar comparison groups
b) Indirect comparisons
c) Dissimilar attention and care
d) Dissimilar expectations or behaviours
e) Dissimilar assessment of outcomes
f) Unreliable assessment of outcomes
g) Lots of people not followed-up
h) Outcomes counted in the wrong group

2.2 Don't be misled by unreliable summaries of treatment comparisons!

a) Unsystematic summaries
b) Selective reporting
c) Unfounded assumptions

2.3 Don't be misled by how treatment effects are described!

a) Just words
b) Relative effects
c) Average effects
d) Few people or events
e) Subgroup analyses
f) Statistically significant
g) No confidence interval
h) No evidence

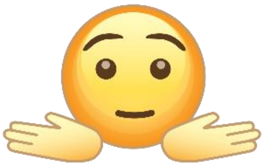

3. Make well-informed treatment choices

Deciding what to do requires judgements about the relevance of the evidence, how important the good and bad outcomes are to you, and how sure you can be about the treatment effects.

3.1 What is the problem and what are the options?

a) What is your health problem and what are your options?

3.2 Is the evidence relevant?

a) What outcomes matter to you?
b) Are the people (or animals) very different from you?
c) Are the treatments different from those available to you?
d) Are the circumstances different from yours?

3.3 Do the advantages outweigh the disadvantages?

a) Do the advantages outweigh the disadvantages for you?
b) How sure are you about the treatment effects?

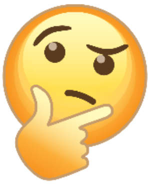

Competences and Dispositions for Informed Health Choices

Competences

To be able to make well-informed decisions about treatments (actions intended to maintain or improve the health of individuals or communities), students should be able to:

1. **Identify claims about the effects of treatments**
   a) Identify when a treatment claim has an untrustworthy basis
   b) Recognise when a claim should be questioned
   c) Recognise when to go from quick to slow thinking about treatment claims
2. **Recognise when evidence used to support a treatment claim is trustworthy or untrustworthy**
   a) Recognise unfair treatment comparisons
   b) Recognise unreliable summaries of treatment comparisons
   c) Recognise misleading presentations of treatment effects
3. **Make well-informed decisions**
   a) Clarify and understand the problem and options when making decisions about treatments
   b) Judge the relevance of evidence used to inform decisions about treatments
   c) Weigh the advantages and disadvantages of treatments, taking into account how important the benefits and harms are, the costs, and the certainty of the evidence
   d) Communicate with others about the advantages and disadvantages of treatments

Dispositions

Students should be disposed to:

1. Be aware of treatment claims and choices
2. Question the basis for treatment claims
3. Go from fast to slow thinking before forming an opinion about a treatment claim, making a claim, or taking a decision
4. Seek evidence to support treatment claims
5. Question the trustworthiness of evidence used to support treatment claims
6. Be alert for misleading presentations of treatment effects
7. Acknowledge and accept uncertainty about the effects of treatments
8. Clarify and understand the problem and options when making decisions about treatments
9. Consider the relevance of the evidence used to inform decisions about treatments
10. Consider the importance of the benefits and harms, the costs, and the certainty of the evidence when making decisions about treatments
11. Make well-informed decisions

Recognise when a treatment claim has an unreliable basis

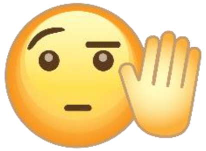

1. Beware of treatment claims like these

We hear claims about the effects of treatments all the time. Many of these are not trustworthy. When you hear someone use one of these reasons to support a claim about the effects of a treatment, you should beware and ask where the evidence is.

Concepts	Short titles	Explanations	Implications
**1.1 Beware of claims that are too good to be true.**			
a) Treatments may be harmful	“100% safe!”	People often exaggerate the benefits of treatments and ignore or downplay potential harms. However, few effective treatments are 100% safe. Similarly, people in need or desperation hope that treatments will work and ignore potential harms. As a result, they may waste time and money on treatments that have never been shown to be useful and may cause harm.	Always consider the possibility that a treatment may have harmful effects.
b) Large, dramatic effects of treatments are rare	“100% effective!”	Large effects (where everyone or nearly everyone treated experiences a benefit or a harm) are easy to detect without fair comparisons, but few treatments have effects that are so large that fair comparisons (designed to reduce the effects of biases and the play of chance) are not needed.	Claims of large effects are likely to be wrong. Expect treatments to have moderate, small or trivial effects, rather than dramatic effects. Do not rely on claims of small or moderate effects of a treatment, which are not based on systematic reviews of fair comparisons of treatments.
c) We can rarely, if ever, be 100% certain about the effects of treatments	“100% certain!”	Fair comparisons of treatments can provide a basis for being confident about the probability of beneficial and harmful effects of treatments. However, it is rarely, if ever, possible to be 100% certain about the size of treatment effects or to know exactly what will happen if a treatment is used. This is especially true for treatments that are intended to prevent something happening a long time in the future. Fair comparisons of such treatments - for example changes in diet or exercise - are difficult, because people need to be followed-up for a very long time and it is difficult to ensure that people adhere to whatever advice they are given. Consequently, claims about the effects of such treatments are often based on associations and explanations. Some people argue that there should be different standards for judgements about the trustworthiness of claims when fair comparisons are difficult. However, it is dishonest not to acknowledge uncertainty, even when there are important limitations on the potential to reduce that uncertainty.	Recognise that there is some uncertainty about the effects of nearly all treatments, and that there is likely to be more uncertainty about some types of treatments. Choices still must be made, but it is better to acknowledge and accept uncertainty than to deny it and make misinformed or poorly informed decisions.
**1.2 Beware of claims based on faulty logic.**			
a) People often recover from illness without treatment	“Treatment needed!”	Effective treatments can prevent health problems and premature death, and improve the quality of life. However, nature is a great healer and people often recover from illness without treatment. Likewise, some health problems may get worse despite treatment, or treatment may actually make things worse. Not using a treatment is not the same as “no treatment”. Waiting to see what happens (“letting nature take its course”), with or without treating symptoms such as pain, is a treatment option.	Always consider the usual course of a health problem when considering treatments other than waiting to see what happens. Sometimes treatment is not needed and may even make things worse.

Concepts	Short titles	Explanations	Implications
b) Beliefs about how treatments work are not reliable predictors of the presence or size of actual effects of treatments	"It works like this!"	Treatments that should work in theory often do not work in practice, or may turn out to be harmful. A plausible explanation of how or why a treatment might work does not prove that it actually does work, or that it is safe. And even if there is plausible evidence that a treatment works in ways likely to be beneficial, the size of any such treatment effect, and its safety, cannot be predicted. For example, most drugs in a class of heart medicines called beta-blockers have beneficial effects in reducing recurrence of heart attacks; but one of the drugs in the class – practolol – caused unpredicted serious complications in patients' eyes and abdomens.	Do not assume that claims about the effects of treatments based on an explanation of how they might work are correct if the treatments have not been assessed in systematic reviews of fair comparisons of treatments.
c) An 'outcome' may be associated with a treatment, but not caused by the treatment	"Associated with!"	The fact that a possible treatment outcome (i.e. a potential benefit or harm) is associated with a treatment does not mean that the treatment caused the outcome. The association or correlation could instead be due to chance or some other underlying factor. For example, people who seek and receive a treatment may be healthier and have better living conditions than those who do not seek and receive the treatment. Therefore, people receiving the treatment might appear to benefit from the treatment, but the difference in outcomes could be because they are healthier and have better living conditions, rather than because of the treatment.	Do not assume that an outcome was caused by a treatment unless other reasons for an association have been ruled out by a fair comparison.
d) More data is not necessarily better data, whatever the source	"Real world data!"	Claims that are based on "big data" (data from large databases) or "real world data" (routinely collected data) can be misleading. More data simply gives a more statistically precise estimate of whatever biases there might be in a treatment comparison that uses routinely collected data. It is only possible to control for confounders that are already known and have been measured when using routinely collected data. Unfortunately, routinely collected data often do not include sufficient data to confidently conclude that any association that is found between a treatment and an outcome means that the treatment caused the outcome. Calling routinely collected data "real world data" suggests that data collected in fair comparisons of treatments do not come from the real world and that routinely collected data are somehow more "real world". Databases of routinely collected data may include a broader spectrum of people than data collected in fair comparisons of treatments that have narrow eligibility criteria. However, routine collection of data is rarely planned to include the information that is needed to ensure a fair comparison.	Do not assume that an association between a treatment and an outcome that is found using "big data" or "real world data" means that the treatment caused the outcome unless other reasons for the association have been ruled out
e) Identifying effects of treatments depends on making comparisons	"No comparison needed!"	Unless a treatment is compared to something else, it is not possible to know what would happen without the treatment, so it is difficult to attribute outcomes to the treatment.	Always ask what the comparisons are when considering claims about the effects of treatments. Claims that are not based on comparisons are not reliable.

Concepts	Short titles	Explanations	Implications
f) The results of single comparisons of treatments can be misleading	“A study shows!”	A single comparison of treatments rarely provides conclusive evidence; and results are often available from other comparisons of the same treatments. Systematic reviews of these other comparisons (replications) may yield different results from those based on the initial studies, and these should help to provide more reliable and precise estimates of treatment differences. Even so, obtaining reliable estimates from treatment comparisons must always consider that important studies may remain unpublished, or inaccessible for other reasons.	The results of single comparisons of treatments can be misleading. Consider all the relevant fair comparisons.
g) Widely used treatments or treatments that have been used for a long time are not necessarily beneficial or safe	“Old is better!”	Treatments that have not been properly evaluated but are widely used or have been used for a long time are often assumed to work. Sometimes, however, they may be unsafe or of doubtful benefit.	Do not assume that treatments are beneficial or safe simply because they are widely used or have been used for a long time, unless this has been shown in systematic reviews of fair comparisons of treatments.
h) New, brand-named, technologically impressive, or more expensive treatments may not be better than available alternatives	“New is better!”	New treatments are often assumed to be better simply because they are new, more expensive, or technologically impressive. However, on average, they are only very slightly likely to be better than other available treatments. Some side effects of treatments, for example, take time to appear and it may not be possible to know whether they will appear without long term follow-up.	Do not assume that a treatment is better or safer simply because it is new, brand-named, expensive, or technologically impressive.
i) Increasing the amount of a treatment does not necessarily increase the benefits of a treatment and may cause harm	“More is better!”	Increasing the dose or amount of a treatment (e.g. how many vitamin pills you take) often increases harms without increasing beneficial effects.	If a treatment is believed to be beneficial, do not assume that more of it is better.
j) Earlier detection of ‘disease’ is not necessarily better	“Early is better!”	People often assume that early detection of disease and ‘treating’ people who are at statistical risk of disease lead to better outcomes. However, screening people to detect disease or treating people at statistical risk of disease is only helpful if two conditions are met. First, there must be an effective treatment. Second, people who are treated before the disease becomes apparent must do better than people who are treated after the disease becomes apparent. Screening and treating people at statistical risk of a disease can lead to overdiagnosis and overtreatment. Screening tests can be inaccurate (e.g. misclassifying people who do not have a disease as if they do have the disease). Screening or treating a statistical risk factor as if it is a ‘disease’ can also cause harm by labelling people as being sick when they are not, and because of side effects of the tests and treatments.	Do not assume that early detection of disease is worthwhile if it has not been assessed in systematic reviews of fair comparisons between people who were screened and people who were not screened.

Concepts	Short titles	Explanations	Implications
k) It is rarely possible to know in advance who will benefit, who will not, and who will be harmed by using a treatment	"Personalised medicine!"	Fair comparisons of treatments usually tell us what happened in a group of people. There are very few, if any treatments that have exactly the same effect on everyone. Usually, in a group of people that use a treatment, there are some who benefit, some who do not, and some who are harmed by the treatment. When you use a treatment, it is not possible to know in advance if you will be one of the people who benefits, one of the people who does not benefit, or one of the people who is harmed. Paradoxically, the only way to know whether "personalised medicine"- customising treatment for individuals works - is to test it in fair comparisons. Unless the customisation is 100% effective and 100% safe, it is still not possible to know in advance who will benefit from "personalised care" and who will not. "Personalised medicine" is not really personalised; it is simply an effort to identify subgroups of people who are most likely to benefit from specific treatments.	Fair treatment comparisons provide the best basis for making well-informed decisions about treatments, but there is almost always some uncertainty about who will benefit, who will not, and who will be harmed.
**1.3 Beware of claims based on trust alone.**			
a) Conflicting interests may result in misleading claims about the effects of treatments	"As advertised!"	People with an interest in promoting a treatment (in addition to wanting to help people) - for example, to make money - may promote treatments by exaggerating benefits and ignoring potential harmful effects. Conversely, people may be opposed to a treatment for a range of reasons, such as cultural practices.	Ask if people making claims that a treatment is effective have conflicting interests. If they do, be careful not to be misled by their claims about the effects of treatments.
b) Personal experiences or anecdotes (stories) are an unreliable basis for assessing the effects of most treatments	"It worked for me!"	People often believe that improvements in a health problem (for example, recovery from a disease) was due to having received a treatment. Similarly, they might believe that an undesirable health outcome was due to having received a treatment. However, the fact that an individual recovered after receiving a treatment does not mean that the treatment caused the improvement, or that other people receiving the same treatment will also improve. The improvement (or undesirable health outcome) might have occurred even without treatment.<br><br>One reason that personal experiences - including a series of personal experiences - are sometimes misleading is that experiences, such as pain, fluctuate and tend to return to a more normal or average level. This is sometimes referred to as "regression to the mean". For example, people often treat symptoms such as pain when they are very bad and would improve anyway without treatment. The same applies to a series of experiences. For example, if there is a spike in the number of traffic crashes someplace, traffic lights may be installed to reduce these. A subsequent reduction may give the appearance that the traffic lights caused this change. However, it is possible that the number of crashes would have returned to a more normal level without the traffic lights.	If an individual improved after receiving a treatment it does not necessarily mean that the treatment caused the improvement, or that other people receiving the same treatment will also improve.
c) Opinions of experts or authorities do not alone provide a reliable basis for judging the benefits and harms of treatments	"Recommended by experts!"	Doctors, researchers, and patients – like anyone else - often disagree about the effects of treatments. This may be because their opinions are not always based on systematic reviews of fair comparisons of treatments. Who makes a treatment claim, how likable they are, or how much experience and expertise they have are not a reliable basis for assessing how reliable their claim is.	Do not rely on the opinions of experts or other authorities about the effects of treatments, unless they have taken account of the results of systematic reviews of fair comparisons of treatments.

Concepts	Short titles	Explanations	Implications
d) Peer-reviewed and published treatment comparisons may not be fair comparisons	"Peer-reviewed!"	Even though a comparison of treatments – whether in a single study or in a review of similar studies - has been published in a prestigious journal, it may not be a fair comparison and the results may not be reliable. Peer review (assessment of a study by others working in the same field) does not guarantee that published studies are reliable. Assessments vary and may not be systematic. Similarly, just because a study is widely publicised does not mean that it is trustworthy.	Always consider whether a published comparison of the effects of treatments is fair and whether the results are reliable. Peer-review is a poor indicator of reliability.

Recognise when evidence from treatment comparisons is trustworthy and when it is not

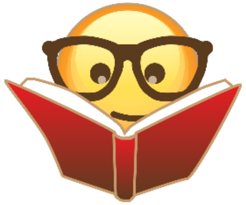

2. Check the evidence from treatment comparisons

A treatment has to be compared to something else to know what would happen without the treatment. For treatment comparisons to be FAIR, the only important difference between comparison groups should be the treatments they receive. Unfair treatment comparisons and unsystematic summaries of treatment comparisons can be misleading. The way that treatment effects are described can also be misleading.

Concepts	Short titles	Explanations	Implications
**2.1 Don't be misled by unfair comparisons!**			
a) Apart from the treatments being compared, the comparison groups need to be similar at the beginning of a comparison (i.e. 'like needs to be compared with like')	Dissimilar comparison groups	If people in the treatment comparison groups differ in ways other than the treatments being compared, the apparent effects of the treatments might reflect those differences rather than actual treatment effects. Differences in the characteristics of the people in the comparison groups at the beginning of the comparison might result in estimates of treatment effects that appear either larger or smaller than they actually are. A method such as allocating people to different treatments by assigning them random numbers (the equivalent of flipping a coin) is the best way to ensure that the groups being compared are similar in terms of both measured and unmeasured characteristics.	Be cautious about relying on the results of non-randomized treatment comparisons (for example, if the people being compared chose which treatment they received). Be particularly cautious when you cannot be confident that the characteristics of the comparison groups were similar. If people were *not* randomly allocated to treatment comparison groups, ask if there were important differences between the groups that might have resulted in the estimates of treatment effects appearing either larger or smaller than they actually are.
b) Indirect comparisons of treatments can be misleading	Indirect comparisons	For many conditions (e.g. depression) there are more than two treatments (for example, different drugs, and different types of psychotherapy). All the possible treatments for a condition are very rarely compared in a single study, so it may be necessary to consider indirect comparisons among treatments. For example, there may be comparisons of drug A with placebo and comparisons of drug B with placebo, but no studies that compare drug A with drug B directly. In this case indirect comparisons among studies may be needed to inform a decision about whether to use drug A or drug B. However, there can be important differences between the studies examined in addition to the treatments they assessed, for example, differences in characteristics of the participants, or the way the comparisons were done, or the outcome measures used. These differences can result in misleading estimates of treatment effects.	Indirect comparisons are sometimes needed to inform treatment choices. When they are, careful consideration should be given to differences between the studies besides the treatments that were compared.

Concepts	Short titles	Explanations	Implications
c) People in the groups being compared need to be cared for similarly (apart from the treatments being compared)	Dissimilar attention and care	Apart from the treatments being compared, people in the treatment comparison groups should otherwise receive similar care. If, for example, people in one group receive more attention and care than people in the comparison group, differences in outcomes could be due to differences in the amount of attention each group received rather than due to the treatments that are being compared. One way of preventing this is to keep providers unaware of ("blind" to) which people have been allocated to which treatment.	Be cautious about relying on the results of treatment comparisons if people in the groups that are being compared were not cared for similarly (apart from the treatments being compared). The results of such comparisons can be misleading.
d) If possible, people should *not* know which of the treatments being compared they are receiving	Dissimilar expectations or behaviours	People in a treatment group may experience improvements (for example, less pain) because they *believe* they are receiving a better treatment, even if the treatment is not actually better (this is called a placebo effect), or because they behave differently (due to knowing which treatment they received, compared to how they otherwise would have behaved). People in a treatment group may also experience harms (for example, more pain) because of their expectations (this is called a nocebo effect). If individuals know that they are receiving a treatment that they believe is better or worse (that is, they are not "blinded"), some or all of the apparent effects of the treatment may be due either to a placebo effect or because the recipients behaved differently.	Be cautious about relying on the results of treatment comparisons if the participants knew which treatment they were receiving because this may have affected their expectations or behaviour. The results of such comparisons can be misleading.
e) Outcomes should be assessed in the same way (fairly) in the treatment groups being compared	Dissimilar assessment of outcomes	If a possible treatment outcome is assessed differently in two comparison groups, differences in that outcome may be due to *how* the outcome was assessed rather than *because* of the treatments received by people in each group. For example, if outcome assessors believe that a particular treatment works and they know which patients have received that treatment, they may be more likely to observe better outcomes in those who have received the treatment. One way of preventing this is to keep outcome assessors unaware of ("blind" to) which people have been allocated to which treatment. This precaution is less important for "objective" outcomes, like death, than for "subjective" outcomes, like pain.	Be cautious about relying on the results of treatment comparisons if outcomes were not assessed in the same way in the different treatment comparison groups. The results of such comparisons can be misleading.
f) Outcomes should be assessed reliably in treatment comparisons	Unreliable assessment of outcomes	Some outcomes are easy to assess, such as births and deaths. Others are more difficult, such as assessing whether someone is depressed or their quality of life. For treatment comparisons to be meaningful, outcomes that are meaningful to people should be assessed using methods that have been shown to be reliable.	Be cautious about relying on the results of treatment comparisons if outcomes were not assessed using methods that have been shown to be reliable.
g) It is important to measure outcomes in *everyone* who was included in the treatment comparison groups	Lots of people not followed-up	People in treatment comparisons who are not followed up to the end of the study may have worse outcomes than those who completed follow up. For example, they may have dropped out because the treatment was not working or because of side effects. If those people are excluded, the findings of the study may be misleading.	Be cautious about relying on the results of treatment comparisons if many people were lost to follow-up, or if there was a big difference between the comparison groups in the percentages of people lost to follow-up. The results of such comparisons can be misleading.

Concepts	Short titles	Explanations	Implications
h) People's outcomes should be counted in the group to which they were allocated	Outcomes counted in the wrong group	Randomized allocation helps to ensure that people in the comparison groups have similar characteristics before they receive treatment. However, people sometimes do not receive or take the treatment allocated to them. The characteristics of such people often differ from those who do take the treatments allocated to them. Excluding from the analysis people who did not receive the treatments allocated to them may mean that like is no longer being compared with like. "Contamination" may lead to an underestimate of treatment differences relative to what would have happened if everyone had received what was intended.	Be cautious about relying on the results of treatment comparisons if patients' outcomes are not counted in the group to which they were allocated. For example, in a comparison of surgery and drug treatments, people who die while waiting for surgery should be counted in the surgery group, even though they did not receive surgery.
**2.2 Don't be misled by unreliable summaries of treatment comparisons!**			
a) Reviews of treatment comparisons that do not use systematic methods can be misleading	Unsystematic summaries	Reviews that do not use systematic methods may result in biased or imprecise estimates of the effects of treatments because the selection of studies for inclusion may be biased, or the methods may result in some studies not being found. In addition, the appraisal of the quality of some studies may be biased, or the synthesis of the results of the selected studies may be inadequate or inappropriate. To avoid these problems, systematic reviews of fair comparisons begin with protocols, which should be registered and searchable in registries such as Prospero. Even reviews that purport to be systematic may not be.	Whenever possible, use up-to-date systematic reviews of fair comparisons inform decisions rather than non-systematic reviews of fair comparisons of treatments.
b) Unpublished results of fair comparisons may result in biased estimates of treatment effects	Selective reporting	Many fair comparisons are never published, and outcomes are sometimes left out. Those that are published are more likely to report favourable results. As a consequence, reliance on published reports alone sometimes results in the beneficial effects of treatments being overestimated and the adverse effects being underestimated. Biased under-reporting of research is a major problem that is far from being solved. It is scientific and ethical malpractice, and wastes research resources. Selective reporting is an important reason why fair comparisons of treatments should have protocols that are registered and searchable in registries such as clinicaltrials.gov.	Be aware of the possibility of biased underreporting of fair comparisons, and whether or not the authors of systematic reviews have addressed this risk
c) Treatment comparisons may be sensitive to assumptions that are made	Unfounded assumptions	Sometimes treatment claims are based on chains of evidence, or models. For example, the effects of using a diagnostic test may depend on how accurate the test is, assumptions about what will be done based on the results of the test, and evidence of the effects of what is done. Similarly, evidence of the effects of public health and health system policies sometimes comes from models that combine different types of studies and assumptions; and assumptions are sometimes made when fair comparisons are combined in systematic reviews. When treatment comparisons depend on assumptions, it is important to consider their basis and to test how sensitive the results are to plausible changes in the assumptions made. For example, a model used to compare the effects of using different diagnostic tests on outcomes that are important to patients might require an assumption about what actions doctors or patients will take based on test results. If that is uncertain, it is important to consider whether changing that assumption has a substantial impact on the estimated difference in outcomes that are important to patients.	Whenever treatment comparisons depend on assumptions, consider whether the assumptions are well-founded and how sensitive the results are to plausible changes in the assumptions that are made.

Concepts	Short titles	Explanations	Implications
**2.3 Don't be misled by how treatment effects are described!**			
a) Verbal descriptions of treatment effects can be misleading	Just words	A treatment effect (a change in outcomes) is a numerical concept, but it is difficult for some people to understand quantitative information about the effects of treatments. Qualitative (verbal) labels may be easier to understand and can be helpful. However, qualitative descriptions of effects mean different things to different people, for example, saying that a treatment will 'slightly reduce', 'reduce', or 'greatly reduce' the likelihood of an undesirable outcome; or that a side effect is 'frequent' or 'rare'. In addition, verbal descriptions of treatments can be manipulative; e.g. promising 'amazing results' or describing treatments as 'natural', implying that they are safe because of that.	A verbal description of a treatment effect can be helpful, but it should be considered together with quantitative information about the size of the effect. Be wary of manipulative use of language in descriptions of treatments.
b) Relative effects of treatments alone can be misleading	Relative effects	Relative effects (for example, the ratio of the probability of an outcome in one treatment group compared with that in a comparison group) are insufficient for judging the importance of the difference (between the frequencies of the outcome). A relative effect may give the impression that a difference is more important than it actually is when the likelihood of the outcome is small to begin with. For example, if a treatment reduces the probability of getting an illness by 50% but also has harms, and the risk of getting the illness is 2 in 100, receiving the treatment may be worthwhile. If, however, the risk of getting the illness is 2 in 10,000, then receiving the treatment may not be worthwhile even though the *relative* effect is the same. The absolute effect of a treatment is likely to vary for people at different baseline risk.	Always consider the absolute effects of treatments – that is, the difference in outcomes between the treatment groups being compared. Do not make a treatment decision based on relative effects alone.
c) Average differences between treatments can be misleading	Average effects	Average effects do not apply to everyone. For outcomes that are assessed using scales (for example, measuring weight, or pain) the difference between the average among people in one treatment group and the average among those in a comparison group may not make it clear how many people experienced a big enough change (for example, in weight or pain) for them to notice it, or that they would regard as important. In addition, many scales are difficult to interpret and are reported in ways that make them meaningless. This includes not reporting the lower and upper 'anchor', for example, if a scale goes from 1 to 10 or 1 to 100; whether higher numbers are good or bad; and whether someone experiencing an improvement of, say, 5 on the scale would barely notice the difference, would consider it a meaningful improvement, or would consider it a large improvement.	When outcomes are assessed using scales, it cannot be assumed that every individual in the treatment comparison groups experienced the average effect. Be wary of differences on scales that are not explained or easily understood.
d) Small studies in which few outcome events occur are usually not informative and the results may be misleading	Few people or events	When there are only few outcome events, differences in outcome frequencies between the treatment comparison groups may easily have occurred by chance and may mistakenly be attributed to differences between the treatments.	Be cautious about relying on the results of treatment comparisons with few outcome events. The results of such comparisons can be misleading.
e) Results for a selected group of people within a systematic review of fair comparisons of treatments can be misleading	Subgroup analyses	Average effects do not apply to everyone. However, comparisons of treatments often report results for selected groups of participants in an effort to assess whether the effect of a treatment is different for different types of people (e.g. men and women or different age groups). These analyses are often poorly planned and reported. Most differential effects suggested by these "subgroup results" are likely to be due to the play of chance and are unlikely to reflect true differences.	Findings based on results for subgroups of people within treatment comparisons may be misleading.

Concepts	Short titles	Explanations	Implications
f) The use of p-values to indicate the probability of something having occurred by chance may be misleading; confidence intervals are more informative	No confidence interval	The observed difference in outcomes is the best estimate of how relatively effective and safe treatments are (or would be, if the comparison were made in many more people). However, because of the play of chance, the true difference may be larger or smaller than this. The confidence interval is the range within which the true difference is likely to lie, after taking into account the play of chance. Although a confidence interval (margin of error) is more informative than a p-value, the latter is often reported. P-values are often misinterpreted to mean that treatments have or do not have important effects.	Understanding a confidence interval may be necessary to understand the reliability of estimates of treatment effects. Whenever possible, consider confidence intervals when assessing estimates of treatment effects. Do not be misled by p-values.
g) Saying that a difference is statistically significant or that it is not statistically significant can be misleading	Statistically significant	"Statistical significance" is often confused with "importance". The cut-off for considering a result as statistically significant is arbitrary, and statistically non-significant results can be either informative (showing that it is very unlikely that a treatment has an important effect) or inconclusive (showing that the relative effects of the treatments compared are uncertain).	Claims that results were significant or non-significant usually mean that they were statistically significant or statistically non-significant. This is not the same as important or not important. Do not be misled by such claims.
h) Lack of evidence of a difference is not the same as evidence of "no difference"	No evidence	Systematic reviews sometimes conclude that there is "no evidence of a difference" when there is uncertainty about the difference between the effects of treatments. This is often misinterpreted as meaning that there is "no difference" between the treatments compared. However, studies can never show that there is "no difference" ("no effect"). They can only rule out, with specific degrees of confidence, differences of a specific size.	Don't be misled by statements of "no difference" between treatments ("no effect"). Consider instead the degree to which it is possible to confidently rule out a difference of a specified size.

Make well-informed treatment choices

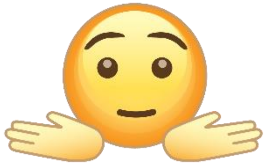

3. Make well-informed treatment choices

Deciding what to do requires judgements about the relevance of the evidence, how important the good and bad outcomes are to you, and how sure you can be about the treatment effects.

Concepts	Short titles	Explanations	Implications
**3.1 What is the problem and what are the options?**			
a) The problem and the treatment options being considered may not be the right ones	What is your problem and what are your options?	Good decisions depend on correctly identifying the problems and considering an appropriate set of options to address the problems. For personal health choices, this means starting with a correct diagnosis (or assessment of risk) and then identifying the treatments that are available. For public health and health system policy decisions, this means describing the problem correctly and identifying the policy options relevant for that problem. Changing how a problem is framed can lead to different options for addressing it.	Make sure you are considering the correct diagnosis or problem, and appropriate options for treating it.

Concepts	Short titles	Explanations	Implications
**3.2 Is the evidence relevant?**			
a) A systematic review of fair comparisons of treatments should report outcomes that are important	What outcomes matter to you?	A fair comparison may not include all outcomes - short and long-term - that are important to you. Patients, professionals and researchers may have different views about which outcomes are important. For example, studies often measure outcomes, such as heart rhythm irregularities, as surrogates for important outcomes, like death after heart attack. However, the effects of treatments on surrogate outcomes often do not provide a reliable indication of the effects on outcomes that are important. Similarly, short-term effects may not reflect long-term effects.	Always consider the possibility that outcomes that are important to you may not have been addressed in fair comparisons. Do not be misled by surrogate outcomes.
b) A systematic review of fair comparisons of treatments in animals or highly selected groups of people may not be relevant	Are the people (or animals) very different from you?	Systematic reviews of studies that only include animals or a selected minority of people may not provide results that are relevant to most people.	Results of systematic reviews of studies in animals or highly selected groups of people may be misleading.
c) The treatments evaluated in fair comparisons may not be relevant or applicable	Are the treatments different from those available to you?	A fair comparison of the effects of a surgical procedure done in a specialised hospital or delivered by an experienced practitioner may not provide a reliable estimate of its effects and safety in other settings, or in the hands of less experienced practitioners. Similarly, comparing a new drug to a drug or dose that is not commonly used (and which may be less effective or safe than those in common use) would not provide a relevant estimate of how the new drug compares to what is commonly done.	Be aware that treatments available to you may be sufficiently different from those in the research studies that the results may not apply to you.
d) Comparisons designed to evaluate whether a treatment can work under ideal circumstances may not reflect what can be expected under usual circumstances.	Are the circumstances different from yours?	Some treatment comparisons are designed to find out if a treatment can work under ideal circumstances, for example, with people who are most likely to benefit, and most likely to comply, and with highly trained practitioners who deliver the treatment exactly as intended. These comparisons, which are sometimes called 'explanatory' or 'efficacy' studies, may not reflect what happens under usual circumstances.	Be aware that the results of studies with the aim of finding out if a treatment can work may overestimate the benefits of a treatment under more usual circumstances.

Concepts	Short titles	Explanations	Implications
**3.3 Do the advantages outweigh the disadvantages?**			
a) Decisions about treatments should not be based on considering only their benefits	Do the advantages outweigh the disadvantages for you?	Decisions about whether or not to use a treatment should be informed by the balance between the potential benefits and the potential harms, costs and other advantages and disadvantages of the treatment. This balance often depends on the baseline risk (the likelihood of an individual experiencing an undesirable event), or on the severity of the symptoms. The balance between the advantages and disadvantages of a treatment is more likely to favour the use of a treatment by people with a higher baseline risk, or more severe symptoms. The balance also depends on how much people value (how much weight they give to) the treatment advantages and disadvantages. Different people may value outcomes differently and sometimes make different decisions because of this. In addition, people usually place more value on things that happen soon than things that happen years into the future. In other words, the further into the future something is (for example, reducing the chance of heart disease or cancer after many years) the more people tend to “discount” its value or importance. The balance between the advantages and disadvantages of treatments may also depend on how much costs and events in the future are discounted.	Always consider the balance between advantages and disadvantages of treatments, taking into consideration the baseline risk or the severity of the symptoms, and what’s important to you.
b) Well done systematic reviews often reveal a lack of relevant evidence, but they provide the best basis for making judgements about the certainty of the evidence	How sure are you about the treatment effects?	The certainty of the evidence (the extent to which the research provides a good indication of the likely effects of treatments) can affect the treatment decisions people make. For example, someone might decide not to use or to pay for a treatment if the certainty of the evidence is low or very low. How certain the evidence is depends on the fairness of the comparisons, the risk of being misled by the play of chance, and how directly relevant the evidence is. Systematic reviews provide the best basis for these judgements and should report an assessment of the certainty of the evidence based on these judgements.	When using the findings of systematic reviews to inform your decisions, always consider the certainty of the evidence.

Glossary

Absolute effects	Absolute effects are differences between outcomes in the groups being compared. For example, if 10% (10 per 100) experience an outcome in one of the treatment comparison groups and 5% (5 per 100) experience that outcome in the other group, the absolute effect is 10% - 5% = a 5% difference.
Allocation	Allocation is the assignment of participants in comparisons of treatments to the different treatments (groups) being compared.
Association or correlation	Association or correlation is a relationship between two attributes, such as using a treatment and experiencing an outcome.
Average difference	The average difference is used to express treatment differences for continuous outcomes, such as weight, blood pressure or pain assessed using a scale. It is the difference between the average value for an outcome measure (for example kilograms) in one group and that in a comparison group.
Baseline risk	Baseline risk is an estimate of the likelihood that an individual or group will experience a health problem before a treatment is used.
Bias	A systematic error that may affect the results of a study because of weaknesses in its design, analysis or reporting
Certainty of the evidence	The certainty of the evidence is an assessment of how good an indication a systematic review provides of the likely effect of a treatment; i.e. the likelihood that the effect will be substantially different from what the studies found (different enough that it might affect a decision). Judgements about the certainty of the evidence are based on factors that reduce the certainty (risk of bias, inconsistency, indirectness, imprecision and publication bias) and factors that increase the certainty.
Chance	In the context of comparisons of treatments, chance is the occurrence of differences between comparison groups that are not due to treatment effects or bias. The play of chance (random error) can lead to incorrect conclusions about treatment effects if too few outcomes occur in studies.
Confidence interval	A confidence interval is a statistical measure of a range within which there is a high probability (usually 95%) that the actual value lies. Wide intervals indicate lower confidence; narrow intervals greater confidence.
Confounders	In treatment comparisons, any factors other than the treatments being compared which may affect the health outcomes being measured
Contamination	Contamination is the inadvertent application of a treatment allocated to one comparison group to people in another comparison group in treatment comparisons.
Data	Information gathered in studies to help address research questions, such as assessing treatment effects
Eligibility criteria	Characteristics used to decide whether people are eligible to participate in a study and should be invited to participate
Explanatory study	An explanatory study (sometimes called an 'efficacy' study) is designed to assess the effects of a treatment given in ideal circumstances, in contrast to a 'pragmatic' study.
Fair comparison	Fair comparisons of treatments are comparisons designed to minimize the risk of systematic errors (biases) and random errors (resulting from the play of chance).
Indirect comparison	A direct comparison is a head-to-head comparison of treatments within a study. If there are no direct comparisons of the treatments of interest, indirect comparisons may be possible - comparisons across studies.
Nocebo effect	An undesirable effect that is or could be caused by an inactive treatment, presumed to act psychologically through suggestion
Outcome	An outcome is a potential benefit or harm of a treatment assessed in a treatment comparison. An outcome measure is how the outcome is assessed in a study.
P-value	A p-value is the probability (ranging from zero to one) that the results observed in a study (or results more extreme) could have occurred by chance if in reality there were no treatment differences.
Placebo	A placebo is a treatment that does not contain active ingredients, which has been designed to be indistinguishable from the active treatment being assessed.
Placebo effect	A measurable, observable, or felt improvement in health or behaviour not attributable to the treatment administered.
Pragmatic study	A pragmatic study (sometimes called an 'effectiveness' study) is designed to assess the effects of a treatment given in the circumstances of everyday practice.
Precision	The extent to which errors due to the play of chance on the results of a study are likely to have occurred
Probability	Probability is the chance or risk of something, such as an outcome, occurring. See Risk
Protocol	The document providing detailed plans for a study
Relative effects	Relative effects are ratios. For example, if the probability of an outcome in the treatment group is 10% (10 per 100) and the probability of that outcome in a comparison group is 5% (5 per 100), the relative effect is 5/10 = 0.50.
Reliable	The reliability of a claim or evidence about a treatment effect is the extent to which it is dependable or can be trusted. It should be noted that reliability often has a different meaning in the context of research, which is the degree to which results obtained by a measurement procedure can be replicated.
Risk	Risk is the probability of an outcome occurring. See Probability

Scale	A scale is an instrument for measuring or rating an outcome with a potentially infinite number of possible values within a given range, such as weight, blood pressure, pain or depression.
Statistical significance	Statistical significance is a difference that is unlikely (below a specified level of confidence – typically 5%) to be explained by the play of chance.
Study	A study is an investigation that uses specified methods to evaluate something. Different types of studies can be used to evaluate the effects of treatments. Some are more reliable than others.
Subgroup	A subgroup is a subdivision of a group of people; a distinct group within a group. For example, in studies or systematic reviews of treatment effects, questions are often asked about whether there are different effects for different subgroups of people in the studies, such as women and men, or people of different ages.
Surrogate outcomes	Surrogate outcomes are outcome measures that are not of direct practical importance but are believed to reflect outcomes that are important. For example, blood pressure is not directly important to patients but it is often used as an outcome in studies because it is a risk factor for stroke and heart attacks.
Systematic review	A systematic review is a summary of research evidence (studies) that uses systematic and explicit methods to summarise the research. It addresses a clearly formulated question using a structured approach to identify, select, and critically appraise relevant studies, and to collect and analyse data from the studies that are included in the review.
Theory	A theory is a supposition or a system of ideas intended to explain something.
Treatment	A treatment is any intervention (action) intended to improve health, including preventive, therapeutic and rehabilitative interventions and public health or health system interventions.
Treatment comparison	Treatment comparisons are studies of the effects of treatments.
