# Supplementary material for: Key Concepts for assessing claims about treatment effects and making well-informed treatment choices
Source: F1000Res. 2019 Jan 23;7:1784. Originally published 2018 Nov 12. [Version 2] doi: 10.12688/f1000research.16771.2 (PMC6290969; doi:10.12688/f1000research.16771.2)
Supplement: Peer review history

# Peer review: Referee response for version 2

**DOI:** 10.5256/f1000research.19687.r43478

**Published:** 2019-01-23

**Authors:** Richard Lehman

**Affiliations:** 1 Institute of Applied Health Sciences, University of Birmingham, Birmingham, UK

No additional comments

I have read this submission. I believe that I have an appropriate level of expertise to confirm that it is of an acceptable scientific standard.

# Peer review: Referee response for version 1

**DOI:** 10.5256/f1000research.18334.r40587

**Published:** 2018-12-13

**Authors:** David Henry, Paulina Stehlik

**Affiliations:** 1 Centre for Research in Evidence Based Practice, Bond University, Gold Coast, Queensland, Australia

**A very useful list and teaching aid but make sure you read the book**

 The Informed Health Choices (IHC) group performs a critical role in creating and regularly updating a framework of concepts that should underpin the decisions that the public make about their health. We can attest to the importance of this initiative having adapted and used some of these concepts in a recent public engagement session on judging the legitimacy of health claims in Australia: ( https://bond.edu.au/researchers/research-bond/research-week-2018/program-and-highlights).

 We strongly support the intention of this program and the sentiments that lie behind it. The education of school children is a particularly innovative and important aspect. The recent categorization of a lengthening list of concepts under Claims, Comparisons and Choices is helpful in aiding understanding of principles and placing them in context.

 Regarding the 2018 update, we raise two general questions about how the key concepts have developed:

 1. Did the authors use rigorous methods to develop the concepts? If not, does it matter?

 2. Is the overall tone too negative? Can healthy skepticism turn into cynicism?

 1. The report is billed as a research article, but the methods used by the authors to develop the IHC concepts are not described in detail. The original set of concepts was based on those identified in the book ‘Testing Treatments’, which itself was a compilation of concepts illustrated by historical examples compiled by the experienced authors. Although the book wasn’t written around a rigorous theoretical framework it is brilliant. It combines key messages (e.g. ‘new is not always better; ‘more is not always better’; ‘earlier is not always better’) with accurate and compelling examples. So, the concepts in the IHC list certainly have face validity. But should the authors have done more? For instance, there is overlap between several of the IHC concepts. Should they have used factor analysis to shorten the list and make it more usable? We think not. They were not designing an instrument to make valid and reliable measurements. Rather, the IHC concepts are a learning resource and their validity has been demonstrated in a randomized trial in children. 

 2. Our concern about the IHC concepts is the generally negative tone. The framing language is always skeptical and to a degree this is understandable and hard to avoid. We are inundated with claims daily and cannot start from a position of equipoise in judging and acting upon those that are relevant to us. The book ‘Testing Treatments’ documents some of the most important advances in medical science, including vaccination, treatment of heart disease, treatment of HIV and some cancers. As the journalist Nick Ross said in a foreword to the first edition of the book “it warmly admires much of what modern medicine has achieved. Its ambitions are always to improve medical practice, not disparage it.” The IHC concept list as a stand alone document lacks this balance. The underlying tone could be interpreted (wrongly) as ‘nothing works’ and ‘be suspicious of all medical claims’. We believe the authors should look for opportunities to use positive framing for some concepts. The preamble to the list should acknowledge the massive progress made by modern medicine and public health, including large reductions in all-cause and some cause-specific mortality rates over the last 50 years. Much of this progress has been made in small increments that individually might not have seemed compelling, but in summation have been dramatic. In our view the main targets of the IHC concepts should be claims that are intentionally misleading, often made by those with vested interests. There are plenty to deal with.

We have read this submission. We believe that we have an appropriate level of expertise to confirm that it is of an acceptable scientific standard.

## References

[1] EvansIThorntonHChalmersIGlasziouP: Testing Treatments: Better Research for Better Healthcare.2006; Reference source 22171402

# Response

**Published:** 2019-01-21

**Authors:** Andrew David Oxman

**Affiliations:** Norwegian Institute of Public Health, Norway

Thank you for these comments.

 1. Did the authors use rigorous methods to develop the concepts? If not, does it matter?

 This is an interesting question, for which we do not believe there is a simple answer. First, we would like to clarify that the IHC Key Concepts provide a framework for developing learning resources, such as those that we tested in randomised trials in Uganda. They are not, by themselves, a learning resource. We believe that we have demonstrated the usefulness of the IHC Key Concepts as a framework for deciding what to teach, for designing learning resources, and for designing a measurement instrument. The results of the randomised trials and process evaluations of those resources suggest that many people have not learned some of these concepts; that they can be learned and used by children and their parents; that people value learning them; and that they help people to think critically about claims. The measurement instrument that we used in the trials was shown to be reliable and valid, and those studies provide some indirect support for both face validity and construct validity for the 13 included concepts (using Rasch analysis). The extent to which very similar concepts have been found to apply across a wide range of different types of interventions provides some additional validation.

 Second, it is unclear how “rigorous methods” should be conceptualised for developing a framework such as that drawing on the IHC Key Concepts. As noted in the Methods section, we are currently conducting a systematic review of related frameworks for critical thinking. We have not so far come across any standard methods for developing frameworks such as these. Our review considers several questions related to the development of frameworks for critical thinking. These include:

- Is there a clear description of the methods that were used?

- Is the basis for the framework clear? For example, was it based on another framework, a model or theory, a systematic review, an unsystematic review, a formal consensus process, or an informal consensus process?

- Are the criteria for including and excluding elements clear?

We believe that we have provided sufficient information about what we have done up to now for others to be able to judge how rigorous or appropriate our methods have been. The basis for developing our framework up to now has been an unsystematic review and an iterative, informal consensus process. A systematic review and a formal consensus process might be considered to be more rigorous, and some might also consider starting with an explicit model or theory to be more rigorous. We are unaware of any evidence suggesting that any of those methods would result in a better framework - however one chooses to define ‘better’.  None of the frameworks that we have reviewed so far have been formally evaluated. Some possible criteria for assessing how sensible a framework is are listed in Box 1. Those are questions that we put to our advisory group (as reported in our first report  of the Key Concepts), and that we have continued to ask others, as reported in this article. For the most part, we have received positive feedback in response to those questions. However, we have received many suggestions for improvements, as reported in this article, and the framework has continued to improve. We could assess more rigorously than we have done up to now the extent to which the Key Concepts are sensible, and  we will consider doing this in future. Something else that we plan to  do more rigorously in future (as noted in the Discussion section) is to systematically summarise the evidence supporting each of the concepts.

 2.  Is the overall tone too negative? Can healthy skepticism turn into cynicism?

 We share this implied concern about the second group of concepts and we will likely reframe at least some of those in the next update. However, we believe the first group of concepts must be framed negatively. The last group of concepts are framed as questions. This same concern came up at a meeting in December at which people from different disciplines discussed the applicability of the IHC Key Concepts to interventions beyond those in health care. This included agricultural, economic, educational, environmental, development, health, informal learning, management, nutritional, planetary health, policing, social welfare, speech and language, and veterinary interventions. We arrived at a list of concepts that participants agreed are relevant to a wide range of interventions. We must reiterate, however, that the Key Concepts by themselves are not a learning resource. We believe that teachers and those developing teaching/learning resources must be aware of this concern, regardless of how the Key Concepts are framed, and that they must be careful to encourage their students to be healthy sceptics without being nihilists.

# Peer review: Referee response for version 1

**DOI:** 10.5256/f1000research.18334.r40589

**Published:** 2018-12-10

**Authors:** Richard Lehman

**Affiliations:** 1 Institute of Applied Health Sciences, University of Birmingham, Birmingham, UK

This description of the development of Key Concepts is clear and comprehensive. It is a real pleasure to read such strong and well-chosen English in a biomedical paper. The authors' sensitivity to language and meaning is also clear from the thoughtful way they have handled suggestions for changes in the wording of Key Concepts themselves.

 I have no suggestions for changes in the text. The concepts themselves are of permanent value and their authors have given the world a model of openness and rigour. It would be good to hear about their dissemination plan as clearly they have been successful in many ways but deserve to reach the widest audience possible.

I have read this submission. I believe that I have an appropriate level of expertise to confirm that it is of an acceptable scientific standard.

# Response

**Published:** 2019-01-21

**Authors:** Andrew David Oxman

**Affiliations:** Norwegian Institute of Public Health, Norway

Thank you.

# Peer review: Referee response for version 1

**DOI:** 10.5256/f1000research.18334.r40591

**Published:** 2018-11-26

**Authors:** Catherine Mathews

**Affiliations:** 1 South African Medical Research Council, Cape Town, South Africa

This is a manuscript describing the refining of the key concepts which will ideally be used as a basis for developing interventions to help people discern between what are true and false healthcare claims. This manuscript describes the methods and rationale for revising the classification system of key concepts, and for revising the key concepts themselves. It also provides the "results": the revised list of key concepts with explanations and presentation format. 

 This is very important work: the IHC Key Concepts have been the foundation of education interventions that are  being actively tested and implemented in several countries in the world, across rich and poor settings.  It is an international initiative to promote critical thinking and evidence-based health care.

 The manuscript describes excellent work, and it is written clearly and articulately. 

 I have the following minor recommendations for improving the manuscript:

1. The authors write that “adaption of the IHC Key Concepts to claims and decisions about other types of interventions (such as educational, economic and environmental interventions), has contributed to the changes we have made."
  However, the authors have not clarified how the changes were informed by considering other types of interventions. I suggest they provide a brief explanation.
2. On page 3, the authors have written: “We initially obtained feedback from 29 members of an international advisory group”. I suggest they name the group in the manuscript, rather than only in the relevant reference.
3. The conclusion of the Abstract could be stronger, by referring to the overall purpose and potential impact of the revised key concepts.

I have read this submission. I believe that I have an appropriate level of expertise to confirm that it is of an acceptable scientific standard.

# Response

**Published:** 2019-01-21

**Authors:** Andrew David Oxman

**Affiliations:** Norwegian Institute of Public Health, Norway

Thank you.

 1. We have clarified that it is feedback from those who have adapted the Key Concepts that has contributed to changes.

 2. The advisory group did not have a formal name.

 3. We edited the conclusion of the Abstract as suggested.
